# Effect of early sexual initiation on early high fertility, termination of pregnancy and child death in Ethiopia using Ethiopian DHS 2000-2016

**DOI:** 10.4314/ahs.v24i2.29

**Published:** 2024-06

**Authors:** Girmatsion Fisseha Abreha, Abiodum O Ilesanmi, Adesina Oladokun, Araya Abrha Medhanyie

**Affiliations:** 1 School of Public Health, Mekelle University, College of Health Sciences, Mekelle, Tigray region, Ethiopia; 2 Department of Obstetrics & Gynecology, University of Ibadan, University College Hospital, Ibadan, Oyo state, Nigeria

**Keywords:** Early sexual intercourse, consequences, Ethiopia

## Abstract

**Background:**

Early sexual initiation is defined as an experience of first sexual intercourse before the age of 18 years. Young girls in sub-Saharan countries initiate sex at early age and are suffering from unintended pregnancy, and related sexual and reproductive health problems.

**Objectives:**

To assess the association of early sexual intercourse with early high birth rate, abortion and under-five child death among young girls in Ethiopia.

**Methods:**

This study used the Ethiopian Demographic Health Survey (EDHS) data from 2000 to 2016. A total of 12,002 sexually active young women aged 15-24 years pool data were used. Binary logistic regression model was used to assess the association between dependent (early sexual initiation) and independent variables and presented using adjusted odd ratio with 95% CI.

**Results:**

Young girls started sexual intercourse as early as 10 years. The young women with high birth rate [4.74, 95% CI (3.53-6.37)], those ever terminated pregnancy [1.77, 95% CI (1.25-2.52)], and had child death history [1.48, 95% CI (1.15-1.91)] were positively associated with early sexual intercourse.

**Conclusion:**

Early sexual initiation among young women is associated with early motherhood, high fertility, child death and poor reproductive health outcomes. Education program on contraceptives and condom accessibility are critical.

## Introduction

An estimated 252 million women aged 15-19 live in developing regions which account for about one-sixth of all women of reproductive age (15-49) in developing regions and one-fifth of reproductive age women in Africa^1^. Young population in Ethiopia shared more than 25% of the total population (105 million) of Ethiopia^2^. However, many young people are suffering by preventable morbidities and mortality mainly due to the sexual and reproductive health problems in developing counties. Young women are more disproportionally affected. There are different reasons for the poor sexual and reproductive health of young women, early sexual initiation is indicated the major causes in developing countries^3^-^8^.

According to different studies, early sexual initiation is defined as an experience of first sexual intercourse before 18 years of age. Early sexual initiations and lack of necessary precautions and knowledge about culture, violence and others among young girls' sexual behavior is one of the major problems in developing countries. Where, it is associated with teenage pregnancy, unsafe abortion, anemia, sexually transmitted infections (STIs), postpartum hemorrhage and mental disorders (depression), preterm and low birth weight^3^-^6^. For this reason, young girls need adequate support and guidance. This might lead to unhealthy choices which will become lifelong habits and affect their future life and development^7^.

In Ethiopia, young girls initiate sexual intercourse as early as 13 and unwanted pregnancy accounts over half of pregnancies to girls under 15. There are higher maternal deaths among young women aged 15-19 which accounts for 17.4% pregnancy related deaths^8^. There are policies and strategies in Ethiopia^9^,^10^. However, there is a gap in the implementation of policies and strategies where many young girls are suffering due to preventable causes. Early sexual intercourse is still higher and not shown any reduction since 2000^8^,^11^. Many studies in Ethiopia focused on risky sexual behaviors^12^-^15^. However, these studies lack to assess the consequences of early sexual intercourse on early motherhood, high birth rate, higher rate of abortion and under-five child mortality among young girls. Therefore, the aim of this study was to assess the peak age at sexual initiation, trend and the association with early motherhood, high birth and abortion rate, and under-five child death among young girls aged 15-24 years in Ethiopia.

## Data and methods

This study used secondary data source from Ethiopian Demographic Health Survey (EDHS) using four DHS data from 2000 to 2016. Data on sexually active young girls' age 15-24 years were used for analysis. The DHS is the nationally representative household survey that is taken from all the nine regions and two administrative cities of Ethiopia. To the current time, a total of five Ethiopian DHS were conducted in Ethiopia (2000, 2005, 2011, 2016, and mini-2019). The Ethiopian DHS 2019 full dataset is not currently available. Therefore, this study used four EDHS datasets. A total sample weight of 12,002 sexually active young girls aged 15-24 years were included in the analysis. The samples included from EDHS 2000 (n=3,295), 2005 (n=2,746), 2011 (n=3,096) and 2016 (n=2,865).

Sexually active young girls are those young girls who had sexual experience before the survey. Early sexual initiation before the age of 18 years among young girls was the dependent variable. Socio-demographic and economic, maternal and reproductive health, and household characteristics were considered as independent variables.

Data were checked for missing values and were cleaned. Data were analyzed using STATA version 14. Sampling weights and individual weights were used to adjust probability distribution and proper representation. Individual weights were used for descriptive statistics and sampling weights were used during regression and chisquare test. Cross-tabulation was done between explanatory variables and the outcome variable. Trend analysis was analyzed to see a change in the age at sexual intercourse between the 2000 and 2016. A binary logistic regression was used to assess the association between early sexual intercourse with high birth rate, child death and abortion rate. Variables with p-value of < 0.05 in multivariable logistic regression model were statistically significant and results were reported with adjusted odds ratio and their respective 95% CI. Goodness of fit of model and Multi-colinearity were checked. On ethical consideration, ethical clearance was obtained from Institutional Review Board of UCH (University College Hospital), University of Ibadan, Nigeria (Ref no: UI/EC/19/0662) and from local in Ethiopia, College of Health Science, Mekelle University. Permission to access the Ethiopian DHS data was obtained from ICF international.

## Result

### Socio-demographic characteristics

Most of the young women included in the survey were from three regions Oromia (37.5%), Amhara (29.7%), and South Ethiopia (15.5%). Majority, 83.3% of young women were from rural residence, 56.3% had no education, and 50% Orthodox Christian followers. There was a significant difference in early sexual intercourse between regions of Ethiopia (Table 1).

**Table 1:** Socio-demographic characteristics of sexually active young women age 15-24 years in the EDHS 2000-2016

Characteristics		Total pool Weighted frequency (%) (n=12,002)	Early sexual initiation Weighted frequency (%) (n=8,564)	Normal age sexual initiation Weighted frequency (%) (n=3,438)	P for difference in socio-demographic by age at sexual initiation
**Age**	15-19	3,839(32.0)	3,400(88.6)	439(11.4)	P<0.0001
	20-24	8,164(68.0)	5,164(63.3)	2,999(36.7)	
**Region**	Tigray	927(7.7)	701(75.6)	226(24.4)	P<0.0001
	Afar	153(1.3)	120(78.1)	34(21.9)	
	Amhara	3,568(29.7)	3,019(86.6)	549(15.4)	
	Oromia	4,497(37.5)	3,017(67.2)	1,481(32.9)	
	Somali	261(2.2)	182(69.6)	79(30.4)	
	Benshangul	149(1.3)	115(77.1)	34(22.9)	
	SNNPR	1,857(15.5)	1,080(58.2)	776(41.8)	
	Gambela	62(0.5)	48(77.2)	14(22.8)	
	Harari	35(0.3)	23(64.7)	12(35.3)	
	Addis Ababa	440(3.7)	229(52.1)	211(47.9)	
	Diredawa	53(0.4)	31(58.3)	22(28.7)	
**Residence**	Urban	2,010(16.8)	1,164(57.9)	846(42.1)	P<0.0001
	Rural	9,992(83.3)	7,400(74.1)	2,592(25.9)	
**Educational level**	No education	6,759(56.3)	5,241(77.5)	1,517(22.4)	P<0.0001
	Primary	3,844(32.0)	2,720(70.7)	1,125(29.2)	
	Secondary	1,047(8.7)	527(50.3)	520(49.7)	
	Higher	352(2.9)	76(21.5)	276(78.5)	
**Religion**	Orthodox	6,002(50.0)	4,489(74.8)	1,513(25.2)	P<0.0001
	Christian				
	Protestant	1,915(16.0)	1,177(61.5)	738(38.5)	
	Muslim	3,743(31.2)	2,682(71.7)	1,061(28.3)	
	Others*	343(2.9)	216(62.9)	127(37.1)	
**Wealth index**	Poorest	2,861(23.8)	2,219(77.5)	643(22.5)	P<0.0001
	Poorer	2,437(20.3)	1,858(76.2)	580(23.8)	
	Middle	2,682(22.4)	2,000(74.6)	682(25.4)	
	Richer	1,892(15.8)	1,289(68.1)	603(31.9)	
	Richest	2,129(17.7)	1,198(56.3)	931(43.7)	
**Marital status**	Never in union	705(5.9)	346(49.0)	360(51.0)	P<0.0001
	Currently in union	9740(81.1)	6,983(71.7)	2,757(28.3)	
	Formerly in union	1557(13.0)	1,235(79.3)	321(20.7)	
**EDHS year**	2000	3,295(27.4)	2,461(74.7)	834(25.3)	P<0.0001
	2005	2,746(22.9)	2,081(77.8)	665(24.2)	
	2011	3,096(25.8)	2,094(67.6)	1,002(32.4)	
	2016	2,865(23.9)	1,928(67.3)	937(32.7)	

Other*= Catholic, traditional and other; SNNP = Southern Nations Nationalities and People's Region

### Sexual and reproductive health characteristics

About 3,345 (41.0%) young women gave birth between the age 15 and 17 years, and 568(7.0%) gave birth at age below 15 years. Most, 6,702(55.8%) young women had given birth to one to two children and 1,457(12.1%) women gave birth to three and more children. One fourth, 1,038 (26.3%) young women had short birth interval (less than 24 months). More than one third, 582 (33.9%) of current pregnancies were unwanted. From the total 7,926 young women who had given birth, 2,579 (32.5%) of the last birth were unwanted. About 749 (6.2%) young women had ever terminated pregnancy and 1,325 (11.0%) had history of child death. Contraceptive use, unmet need and STIs were higher among the young women who start sex at early age compared to those who start sex at normal age. There were also a significant difference in having a number of children (p<0.0001), early birth (p<0.0001), short birth interval (p<0.0001), caesarean delivery (p<0.0001), child death history (p<0.0001), being anemic and under-weight among those who initiate sex at early age than those who started sex at normal age (Table 2).

**Table 2:** Reproductive characteristics of young women age 15-24 years in the EDHS 2000-2016

Variables	Total Weighted frequency (%) (n=12,02)	Early sexual initiation Weighted frequency (%) (n=8,564)	Normal age sexual initiation Weighted frequency (%) (n=3,438)	P for difference in reproductive characteristics by age at sexual initiation
**Child ever born**				P<0.0001
**no**	3,843(32.0)	2,342(60.9)	1,500(39.1)	
**1-2 children born**	6,702(55.8)	4,835(72.1)	1,868(27.9)	
**>=3 children born**	1,457(12.1)	1,387(95.2)	70(4.8)	
**Age at first birth**				P<0.0001
**<15 years**	568(7.0)	566(99.6)	2(0.4)	
**15-17 years**	3,345(41.0)	3312(99.0)	33(1.0)	
**>=18 years**	4,246(52.0)	2343(55.2)	1,903(44.8)	
**Total birth in the last five years**				P<0.0001
**No births**	4,076(34.0)	2,570(63.1)	1,506(36.9)	
**1-2 births**	7,493(62.4)	5,605(74.8)	1,888(25.2)	
**>=3 births**	434(3.6)	389(89.6)	45(10.4)	
**Preceding birth interval**				P<0.0001
**<24 months**	1,038(26.3)	828(79.8)	210(20.2)	
**>= 24 months**	2,906(73.7)	2,580(88.8)	326(11.2)	
**Currently pregnant**				P=0.0067
**Yes**	1,705(14.2)	1,149(67.4)	556(32.6)	
**No**	10,297(85.8)	7,414(72.0)	2,883(28.0)	
**Current pregnancy wanted**				
**Wanted**	1,132(66.1)	749(66.1)	383(33.9)	P=0.339
**Unwanted**	582(33.9)	407(69.9)	175(30.1)	
**Last (birth) child unwanted**				P=0.928
**Wanted**	5,347(67.5)	4,037(75.5)	1,311(24.5)	
**Unwanted**	2,579(32.5)	1,957(75.9)	622(24.1)	
**Ever had terminated pregnancy**				P=0.0007
**No**	11,253(93.8)	7,971(70.8)	3,282(29.2)	
**Yes**	749(6.2)	593(79.1)	157(20.9)	
**Last birth cesarean section**				P<0.0001
**Yes**	132(1.7)	80(60.8)	52(39.2)	
**No**	7,793(98.3)	5,912(75.9)	1,881(24.1)	
**Currently breast feeding**				P<0.0001
**Yes**	5,770(48.1)	4,308(74.7)	1,463(25.3)	
**No**	6,232(51.9)	4,256(68.3)	1,976(31.7)	
**Child death history**				P<0.0001
**Yes**	1,325(11.0)	1,147(86.6)	178(13.4)	
**No**	10,677(89.0)	7,417(69.5)	3,260(30.5)	
**Currently contraceptive use**				P<0.0001
**Yes**	2,471(20.6)	1,604(64.9)	868(35.1)	
**No**	9,531(79.4)	6,960(73.0)	2,571(27.0)	
**Unmet need for contraception**				P<0.0001
**Infecund, menopausal**	2,101(17.5)	1,571(74.8)	529(25.2)	
**Unmet need**	2,991(24.9)	2,233(74.6)	759(25.4)	
**Currently using contraceptives**	2,471(20.6)	1,604(64.9)	868(35.1)	
**No unmet need**	4,439(37.0)	3,156(71.1)	1,283(28.9)	
**STI in the last 12 months**				P=0.1535
**Yes**	827(6.9)	616(74.5)	211(25.2)	
**No**	11,175(93.1)	7,948(71.1)	3,227(28.9)	
**Anemic status of women**				P<0.0001
**Normal level**	5,662(78.6)	3,854(68.1)	1,808(311.9)	
**Anemic**	1,540(21.4)	1,116(72.5)	423(27.5)	
**ANC visit during pregnancy**				P<0.0001
**Yes**	3,369(42.5)	2,420(71.8)	949(28.2)	
**No**	4,557(57.5)	3,574(78.4)	984(21.6)	
**Place of delivery**				P<0.0001
**Home**	6,623(83.6)	5,149(77.7)	1,474(22.3)	
**Health facility**	1,304(16.5)	845(64.8)	459(35.2)	
**History of perceived small weight child birth**				P<0.0001
**Average and above**	8,766(73.0)	6,016(68.6)	2,750(31.4)	
**Smaller than average**	3,236(27.0)	2,548(78.7)	688(21.3)	
**Experience of obstetric fistula**				P=0.105
**Yes**	34(1.0)	26(75.0)	9(25.0)	
**No**	3,349(99.0)	2,242(66.9)	1,107(33.1)	

### Trend in Early sexual intercourse

**Figure 1:**
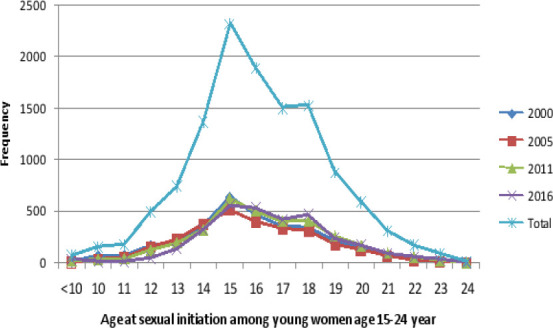
Age at sexual initiation among young women age (15-24 years) in Ethiopia, EDHS 2000-2016

The young girls started sexual activites as early as 10 years and less in Ethiopia. The peak age at sexual initiation was between 14 and 15 years (first phase). However, the average median age at sexual initiation was 16 years (IQR:14-18). There were slight variations in age at sexual initiation in the Ethiopian DHS 2016 where many girls started sex at age between 17 and 18 years. The age between 17 and 18 years can be considered as the second phase to start sex for young girls (Fig 1). Most, young girls started sex at early age 71% (8,719/12,278) and higher during Ethiopia DHS 2000 which was 26.3% (2,340/8,719) and lower during Ethiopia DHS 2016 which was 23.5% (2,051/8,719).

### Association between early sexual intercourse with early high birth and abortion rate, and child death

Variables at bi-variate with p-value less than 0.05 were considered for multivariate logistic regression model. The young women who have greater and equal to three children ever born [4.74, 95% CI (3.53-6.37)], those ever had terminated pregnancy [1.77, 95% CI (1.25-2.52)], and have child death history [1.48, 95% CI (1.15-1.91)] were positively associated with early sexual initiation compared to those young girl girls who started sex at normal age. Moreover, young girls who started sex at early age were more likely to be underweight [1.50, 95% CI (1.06-2.12)], short in stature (<150cm) [1.53, 95% CI (1.20-1.95)], and having history of homebirth [1.55, 95% CI (1.3-1.84)] compared to those who start sex at normal age (Table 3).

**Table 3:** Association between early sexual initiation, and poor maternal and child health outcomes among sexually active young women age 15-24 year in the EDHS 2000-2016

Variables	Early sexual initiation Weighted frequency (%) (n=8,564)	Normal age at sexual initiation Weighted frequency (%) (n=3,438)	Odds ratio (95% CI)	Adjusted odds ratio (95% CI)
**Child ever born**				
**Less than 2 children**	7,177 (68.1)	3,368 (31.9)	-	-
**>=3 children born**	1,387 (95.2)	70 (4.8)	7.1 (5.67-8.91)^a^	4.74 (3.53- 6.37)^a^
**Ever had terminated pregnancy**				
**Yes**	593 (79.1)	157 (20.9)	1.45 (1.21-1.73)^a^	1.77 (1.25-2.52)^a^
**No**	7,971 (70.8)	3,282 (29.2)	-	-
**Last birth cesarean section**				
**Yes**	80 (60.8)	52 (39.2)	0.47 (0.35-0.61)^a^	0.75 (0.53-1.08)
**No**	5,912 (75.9)	1,881 (24.1)	-	-
**Child death history**				
**Yes**	1,147 (86.6)	178 (13.4)	2.93 (2.47-3.46)^a^	1.48 (1.15-1.91)^b^
**No**	7,417 (69.5)	3,260 (30.5)	-	-
**History of small birth weight child**				
**Smaller than average**	2,548 (78.7)	688 (21.3)	1.76 (1.6-1.94)^a^	1.1 (0.94-1.26)
**Average and above**	6,016 (68.6)	2,750 (31.4)	-	-
**Anemic status of women**				
**Anemic**	1,116 (72.5)	423 (27.5)	1.23 (1.1-1.38)^a^	0.97 (0.83-1.14)
**Normal level**	3,854 (68.1)	1,808 (311.9)	-	-
**Stature of women**				
**<150cm**	1,078 (76.1)	339 (23.9)	1.44 (1.24-1.66)^a^	1.53 (1.20-1.95)^a^
**150 cm**	4,408 (72.1)	1,673 (27.5)	1.30 (1.19 1.43)^a^	1.3 (1.12-1.51)^a^
**>=160cm**	1,899 (64.8)	1,030 (35.2)	-	-
**Nutritional status of young women**				
**Under weight**	1,671 (76.5)	515(23.5)	2.23 (1.83-2.72)^a^	1.50 (1.06-2.12)^c^
**Normal**	5,480 (70.0)	2,346(30.0)	1.67 (1.39-2.01)^a^	1.4 (0.89-1.72)
**Over weight**	228 (57.1)	172(42.9)	-	-
**Currently contraceptive use**				
**Yes**	1,604 (64.9)	868 (35.1)	0.68 (0.62-0.749)^a^	0.90 (0.76-1.05)
**No**	6,960 (73.0)	2,571 (27.0)	-	-
**ANC visit during pregnancy**				
**Yes**	2,420 (71.8)	949 (28.2)	0.70 (0.63-0.78)^a^	0.91 (0.78-1.07)
**No**	3,574 (78.4)	984 (21.6)	-	-
**Place of delivery for recent birth**				
**Home**	5,149 (77.7)	1,474 (22.3)	1.94 (1.73-2.19)^a^	1.55 (1.3-1.84)^a^
**Health facility**	845 (64.8)	459 (35.2)	-	-

a significant at p-value <0.001

b significant at 0.001-0.01

c significant at 0.02-0.04

## Discussions

The aim of this study was to assess the peak age at sexual initiation and the association with early motherhood, high fertility rate, termination of pregnancy and child death among young girls age 15-24 years. Our finding showed that, most (71%), young girls start sex at early age. The peak age at sexual initiation was between 14 and 15 years which can be considered as first phase followed by second phase at age between 17 and 18 years. The phases are the age at which most women start sexual intercourse. High birth rate (>=3 children), ever had terminated pregnancy and have child death history were positively associated with early sexual intercourse compared to those young girls who started sex at normal age.

According to the current findings, the peak age to start sexual intercourse was at age 14 to 15 years which is too early indicating many young girls are involved in sexual activities at teen age which may expose them to different sexual and reproductive health problems like unwanted pregnancy, abortion, complications of abortion, STIs, HIV, and other problems^16^-^19^. This condition in Africa or developing countries may have lifelong effect on young girls which leads them to pregnancy and childbirth related complications such as prolonged labour, bleeding, fistula and maternal deaths, and further disruption of schooling, dropout from school, and economical dependency. Therefore, focusing on peak age and interventions such as safe sex practice and school and out school reproductive health education and service is services are needed with friendly approach. Additionally, monitoring and evaluation of youth health programs is important.

The odd of experiencing early sexual initiation was more likely associated among the young girls who have high birth rate (three and more children) in this study. This indicates, if young girls start sex at early age, the probability of getting pregnant and early childbirth is higher^20^. their reproductive life span will be longer, the probability of giving birth to too many children will be higher especially in developing countries and rural setting^21^,^22^. This means, early sexual initiation is leading young girls to have many children. This may be due poor awareness and poor contraceptives utilization among young girls 19,22,23. Therefore, in order to reduce high fertility at early age, there is a need to give focus on early sexual initiation mechanism of delaying pregnancy. Moreover, a special way of reaching young married women in developing countries like expanding modern contraceptives and youth friendly services is needed.

In this study, ever had abortion was higher among young girls who started sex at early age. This indicates young girls are experiencing high-rate abortion as result of early sexual initiation. The risky sexual behavior of the young girls, poor awareness about condom and other contraceptives may leads unwanted pregnancies and abortions among young girls,^23^. As evidences indicated, bleeding due to unsafe abotions which is the cause of maternal death in developing countries including in Ethiopia^24^. Therefore, modernizing abortion, post abortion services and programs that address accessibility of contraceptive for young girls are needed.

The odd of experiencing child death history was associated with early sexual intercourse among the youth women in this study. This indicates that young girls who initiated sex at early age are becoming pregnant and giving birth as teenagers which is associated with child death. This is supported by studies where early sexual initiation and early marriage leads to high child morbidity and mortality^4^,^25^. This could be due to inadequate knowledge on child care, poor feeding practice practices and not using maternal health services.^19^ In this study, most of the pregnant young women gave birth at home. Unclean delivery may expose newborn to infection and early newborn death. Focus on maternal health service use like antenatal care and institutional birth should be encouraged among youth women. In addition, emphasis is also important on educating young mothers on how to care for a newborn during postnatal period and home visits at community level.

As strength, this study used large sample of young girls which is representative using data from Ethiopian DHS 2000 to 2016. As limitation, anemic status of girls was based on three DHS data; 2005, 2011 and 2016. DHS 2000 has no data related to anemia status of girls. Experience of leakage of urine and faces (obstetric fistula) data were based on the EDHS 2005 and 2016 data due to since we did not get data related to obstetric fistula in EDHS 2000 and 2011. Therefore, it is better to consider these limitations in interpretation of the findings.

## Conclusions and recommendations

The level of early sexual intercourse at teenage is very higher among young girls age 15-24 years. The peak age at sexual initiation is between 14 and 15 (first phase) followed by second phase at age between 17 and 18 years. High birth rate, ever had abortion and having child death history is positively associated with early sexual intercourse compared to those young girls who started sex at normal age. Therefore, education program on how to care for a newborn during pregnancy, postnatal period and home visits at community level is critical. Moreover, strengthening sexual reproductive health education, youth friendly service, accessibility of contraceptives, maternal health service and outreach program at rural settings are important to reduce the problems.
